# Supplementary material for: Halogens in Acetophenones Direct the Hydrogen Bond Docking Preference of Phenol via Stacking Interactions
Source: Molecules. 2021 Aug 12;26(16):4883. doi: 10.3390/molecules26164883 (PMC8400467; doi:10.3390/molecules26164883)
Supplement: Supplementary file 1 [file molecules-26-04883-s001.zip › molecules-1326346-SI.pdf]

Supporting Information:

Halogens in acetophenones direct the hydrogen bond docking preference of phenol via stacking interactions

C. Zimmermann, M. Lange, M. A. Suhm*

E-mail: msuhm@gwdg.de

Theoretical results

Table S1: ORCA 4.2.1[S1] and Turbomole[S2,S3] keywords used for the different electronic structure optimizations, reaction path optimizations (superscript RP) and relaxed scans (superscript RS).

Level of approximation	Employed keywords
B3LYP-D3(BJ,ABC)/def2-TZVP (ORCA)^{RS}	B3LYP D3BJ def2-TZVP abc grid5 NoFinalGrid UseSym VERYTIGHTSCF TIGHTOPT FREQ
B3LYP-D3(BJ,ABC)/def2-QZVP (ORCA)	B3LYP D3BJ def2-QZVPP abc grid5 NoFinalGrid UseSym VERYTIGHTSCF TIGHTOPT FREQ
B3LYP-D3(BJ,ABC)/ma-def2-QZVP (ORCA)	B3LYP D3BJ def2-QZVPP abc grid5 NoFinalGrid UseSym VERYTIGHTSCF TIGHTOPT FREQ
B3LYP-D3(BJ,ABC)/def2-TZVP (TURBOMOLE)^{RP,RS}	b3-lyp def2-mTZVP grid m5 disp3 bj abc ri

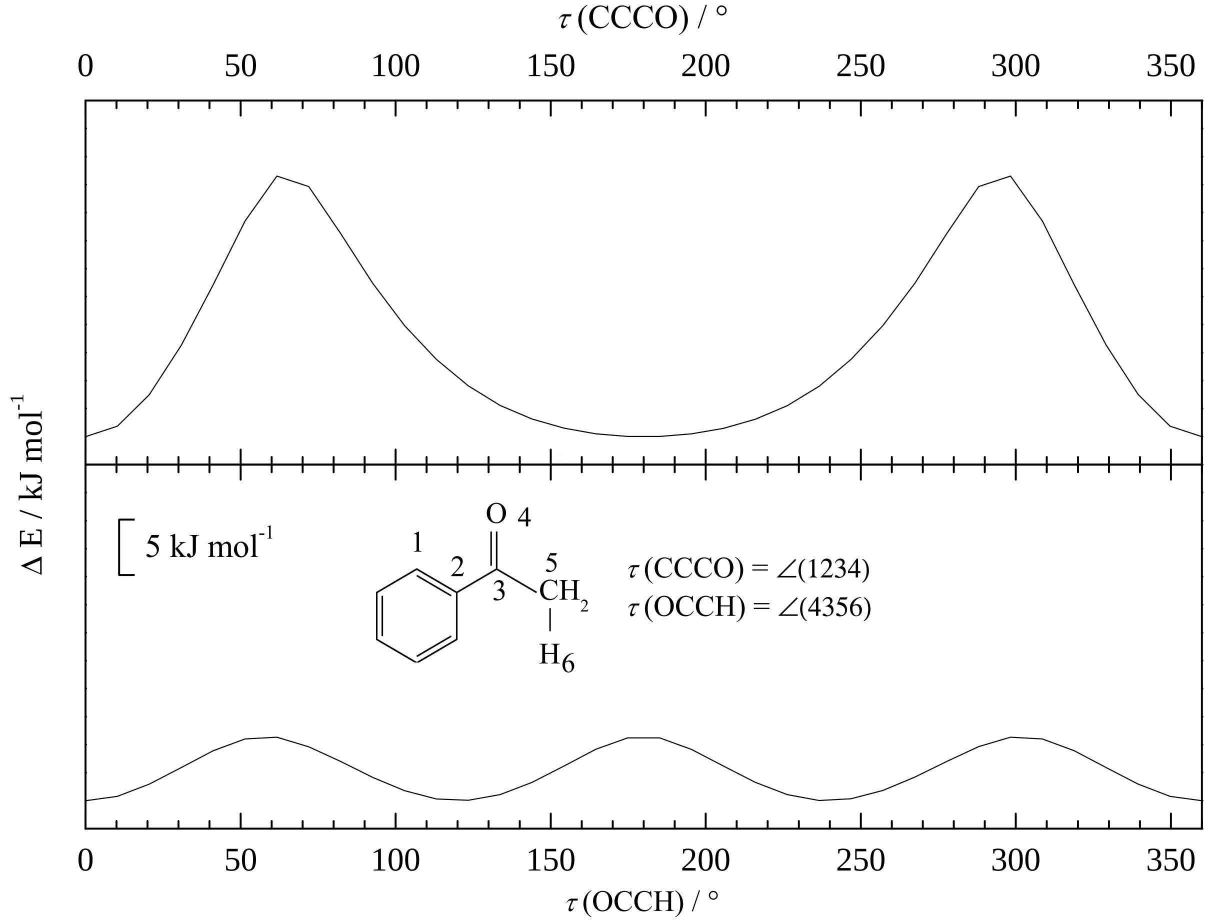

Fig. S1: Relaxed B3LYP-D3(BJ,ABC)/def2-TZVP scans along the aromatic CCCO (upper panel) torsional angle (with equivalent minima at $0°$ and $180°$) and aliphatic OCCH (lower panel) torsional angle for acetophenone (with equivalent minima at $0°$, $120°$, $240°$). Barriers below about $5\,\mathrm{kJ\,mol^{-1}}$ (see insert for the scale) can be largely overcome in jet expansions. Aromatic torsion isomers will likely freeze in their room temperature distribution. Therefore, *meta*-substitution of acetophenone is avoided in this work, whereas *ortho*-substitution is likely to lead to a strong isomer imbalance already at room temperature.

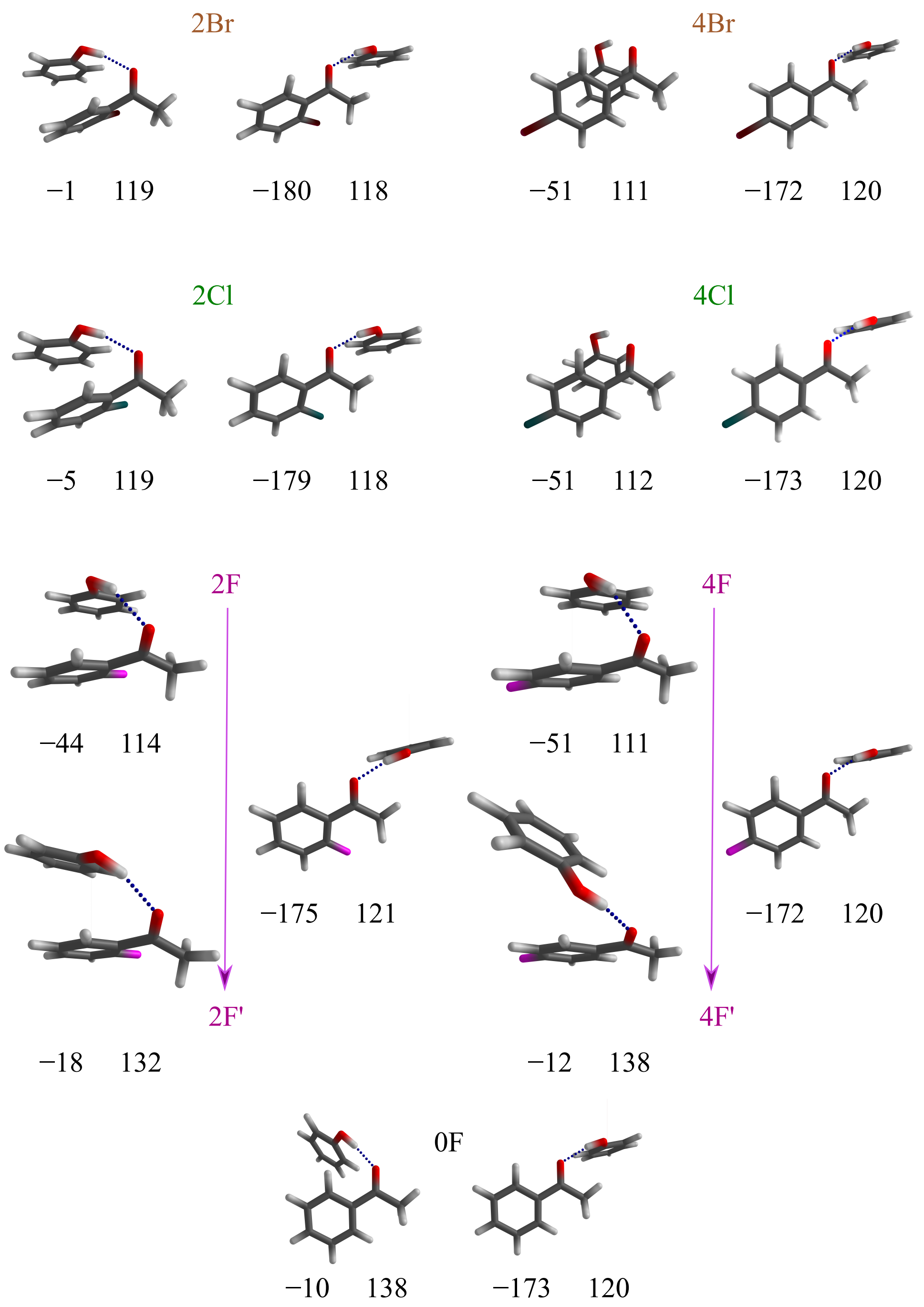

Fig. S2: Structures of the most stable phenol-acetophenone derivative dimers at B3LYP-D3(BJ,ABC)/def2-TZVP level. Additionally, for 2F and 4F the next higher lying isomer for phenyl docking (2F’ and 4F’, with less pronounced stacking) is shown. Also given is the torsional angle τ of the hydrogen-bonded H relative to the phenyl group around the C=O group (C-C=O$\cdots$H, left value; the uniformly negative sign indicates that the solvating phenol is pointing away from the reader) and the hydrogen bond angle α (C=O$\cdots$H, right value), both in °. Methyl side docking shows a uniform angle close to 120° and only slight torsion out of the carbonyl plane. Phenyl side docking is more diverse. All halogenated derivatives stay below 120° in their predicted most stable structures by aromatic stacking. For all but 2Br and 2Cl (where the bulky halogen already tilts the monomer out of the carbonyl plane), this requires substantial out-of-plane torsion of the bridging hydrogen. 0F, 2F’ and 4F’ minimize this torsion by adopting a larger bond angle α, thus forming an *ortho* C-H contact with the phenyl oxygen. The F/F’ isomerism thus represents a trade off involving primary and secondary hydrogen bonds vs. aromatic stacking, with strong spectroscopic consequences.

Table S2: Values for the hydrogen bond angle α between the ketone group and the hydrogen bonded H, and the associated H/Ph torsional angle τ around the ketone group in °. The negative sign for τ corresponds to Fig. S2, where the alcohol substituent is pointing away from the reader. The strong similarity between the standard and augmented basis sets indicates that the structural isomerism discussed in Fig. S2 is robust with respect to basis set extension.

			Ph		Me	
Acceptor	Method	Basis set	τ	α	τ	α
2Br	B3LYP-D3(BJ)	def2-TZVP	-1	119	-180	118
2Br	B3LYP-D3(BJ)	ma-def2-TZVP	-1	119	-179	118
2Cl	B3LYP-D3(BJ)	def2-TZVP	-5	119	-179	118
2Cl	B3LYP-D3(BJ)	ma-def2-TZVP	-5	120	-179	119
2F	B3LYP-D3(BJ)	def2-TZVP	-44	114	-175	121
2F	B3LYP-D3(BJ)	ma-def2-TZVP	-44	114	-176	121
2F’	B3LYP-D3(BJ)	def2-TZVP	-18	132	-	-
2F’	B3LYP-D3(BJ)	ma-def2-TZVP	-18	132	-	-
4Br	B3LYP-D3(BJ)	def2-TZVP	-51	111	-172	120
4Br	B3LYP-D3(BJ)	ma-def2-TZVP	-51	112	-174	120
4Cl	B3LYP-D3(BJ)	def2-TZVP	-51	112	-173	120
4Cl	B3LYP-D3(BJ)	ma-def2-TZVP	-51	112	-174	120
4F	B3LYP-D3(BJ)	def2-TZVP	-51	111	-173	120
4F	B3LYP-D3(BJ)	ma-def2-TZVP	-52	112	-173	120
4F’	B3LYP-D3(BJ)	def2-TZVP	-12	138	-	-
4F’	B3LYP-D3(BJ)	ma-def2-TZVP	-13	138	-	-
0F	B3LYP-D3(BJ)	def2-TZVP	-10	138	-173	120
0F	B3LYP-D3(BJ)	ma-def2-TZVP	-11	138	-173	120

Table S3: Absolute values for the CCC=O phenyl dihedral angle τ in the monomer ketone ($\tau_{\mathrm{mono}}^{\mathrm{ketone}}$), in the methyl sided dimer structure ($\tau_{\mathrm{Me}}^{\mathrm{ketone}}$), and in the phenyl sided dimer structure ($\tau_{\mathrm{Ph}}^{\mathrm{ketone}}$). Methyl docking is seen to have a minor influence (<6°) on the phenyl torsion, whereas phenyl docking has a larger influence (up to 30°, in particular for 2Br, 2Cl, 2F) with stacking as the likely driving force.

Acceptor	Method	Basis set	$\tau_{\mathrm{mono}}^{\mathrm{ketone}}$	$\tau_{\mathrm{Me}}^{\mathrm{ketone}}$	$\tau_{\mathrm{Ph}}^{\mathrm{ketone}}$
2Br	B3LYP-D3(BJ)	def2-TZVP	37	41	62
2Br	B3LYP-D3(BJ)	ma-def2-TZVP	38	42	62
2Cl	B3LYP-D3(BJ)	def2-TZVP	31	35	60
2Cl	B3LYP-D3(BJ)	ma-def2-TZVP	33	35	60
2F	B3LYP-D3(BJ)	def2-TZVP	0	5	25
2F	B3LYP-D3(BJ)	ma-def2-TZVP	0	6	25
2F’	B3LYP-D3(BJ)	def2-TZVP	0	5	27
2F’	B3LYP-D3(BJ)	ma-def2-TZVP	0	6	28
4Br	B3LYP-D3(BJ)	def2-TZVP	0	2	18
4Br	B3LYP-D3(BJ)	ma-def2-TZVP	0	2	19
4Cl	B3LYP-D3(BJ)	def2-TZVP	0	2	18
4Cl	B3LYP-D3(BJ)	ma-def2-TZVP	0	2	18
4F	B3LYP-D3(BJ)	def2-TZVP	0	2	18
4F	B3LYP-D3(BJ)	ma-def2-TZVP	0	2	17
4F’	B3LYP-D3(BJ)	def2-TZVP	0	2	10
4F’	B3LYP-D3(BJ)	ma-def2-TZVP	0	2	11
0F	B3LYP-D3(BJ)	def2-TZVP	0	2	11
0F	B3LYP-D3(BJ)	ma-def2-TZVP	0	2	12

Table S4: Docking preference of phenol complexes with halogenated acetophenone derivatives predicted by B3LYP in $\mathrm{kJ\,mol^{-1}}$ relative to the phenyl side. ΔE^0 includes the harmonically approximated zero-point energy and ΔE^{el} excludes it. Negative values indicate a higher stability of the complex where the solvent docks on the phenyl side of the acetophenone derivative. Due to numerical effects with ma-def2-TZVP leading to single imaginary wavenumbers $<10\,\mathrm{cm^{-1}}$, the energy values for 4Br and 4Cl (*) should be attributed an additional error bar of $0.1\,\mathrm{kJ\,mol^{-1}}$. The last column lists the difference between the electronic (el) and ZPVE-corrected energy differences ($\Delta(\mathrm{el}-0)$) as an indicator how much the hydrogen bond environment is distorted due to stacking. For 0F, 2F', 4F', the hydrogen bond environment is balanced for both docking sides within $0.5\,\mathrm{kJ\,mol^{-1}}$ (a design principle of these carbonyl balances), for the others, phenyl docking profits additionally from hydrogen bond and thus ZPVE weakening by up to $1.3\,\mathrm{kJ\,mol^{-1}}$.

Acceptor	Method	Basis set	$\Delta E^{\mathrm{el}}_{\mathrm{Ph-Me}}$	$\Delta E^{0}_{\mathrm{Ph-Me}}$	$\Delta(\mathrm{el}-0)$
2Br	B3LYP-D3(BJ)	def2-TZVP	−8.52	−9.59	+1.08
2Br	B3LYP-D3(BJ)	ma-def2-TZVP	−8.88	−9.59	+0.71
2Cl	B3LYP-D3(BJ)	def2-TZVP	−6.57	−7.88	+1.30
2Cl	B3LYP-D3(BJ)	ma-def2-TZVP	−6.80	−7.71	+0.91
2F	B3LYP-D3(BJ)	def2-TZVP	−1.15	−1.83	+0.69
2F	B3LYP-D3(BJ)	ma-def2-TZVP	−1.42	−1.75	+0.33
2F'	B3LYP-D3(BJ)	def2-TZVP	−1.18	−1.62	+0.44
2F'	B3LYP-D3(BJ)	ma-def2-TZVP	−1.80	−1.84	+0.04
4Br	B3LYP-D3(BJ)	def2-TZVP	−0.94	−1.68	+0.74
4Br	B3LYP-D3(BJ)	ma-def2-TZVP	−1.51*	−1.92*	+0.41*
4Cl	B3LYP-D3(BJ)	def2-TZVP	−0.41	−1.22	+0.81
4Cl	B3LYP-D3(BJ)	ma-def2-TZVP	−1.00*	−1.47*	+0.47*
4F	B3LYP-D3(BJ)	def2-TZVP	+0.74	−0.12	+0.86
4F	B3LYP-D3(BJ)	ma-def2-TZVP	+0.21	−0.29	+0.50
4F'	B3LYP-D3(BJ)	def2-TZVP	+0.41	+0.36	+0.05
4F'	B3LYP-D3(BJ)	ma-def2-TZVP	+0.10	+0.24	−0.14
0F	B3LYP-D3(BJ)	def2-TZVP	+0.88	+0.75	+0.12
0F	B3LYP-D3(BJ)	ma-def2-TZVP	+0.55	+0.64	−0.09

Table S5: Docking effect on the harmonic OH stretching wavenumbers ω and resulting wavenumber shifts $\Delta\omega$ for methyl (Me) and phenyl (Ph) docking variants of phenol-acetophenone derivative complexes in cm^{-1}. Positive signs indicate an universally higher wavenumber for phenyl docking. Stacking of the aromatic rings for phenyl docking correlates with harmonic shifts from the methyl docking side of $100\,\mathrm{cm}^{-1}$ and more due to the competition with hydrogen bonding.

Acceptor	Method	Basis set	ω_{Me}	ω_{Ph}	$\Delta\omega_{\mathrm{Ph-Me}}$
2Br	B3LYP-D3(BJ)	def2-TZVP	3520	3563	+43
2Br	B3LYP-D3(BJ)	ma-def2-TZVP	3522	3563	+41
2Cl	B3LYP-D3(BJ)	def2-TZVP	3517	3562	+45
2Cl	B3LYP-D3(BJ)	ma-def2-TZVP	3518	3560	+43
2F	B3LYP-D3(BJ)	def2-TZVP	3513	3611	+99
2F	B3LYP-D3(BJ)	ma-def2-TZVP	3513	3613	+101
2F'	B3LYP-D3(BJ)	def2-TZVP	3513	3569	+57
2F'	B3LYP-D3(BJ)	ma-def2-TZVP	3513	3569	+57
4Br	B3LYP-D3(BJ)	def2-TZVP	3510	3649	+139
4Br	B3LYP-D3(BJ)	ma-def2-TZVP	3508	3649	+141
4Cl	B3LYP-D3(BJ)	def2-TZVP	3508	3646	+138
4Cl	B3LYP-D3(BJ)	ma-def2-TZVP	3505	3649	+144
4F	B3LYP-D3(BJ)	def2-TZVP	3502	3635	+133
4F	B3LYP-D3(BJ)	ma-def2-TZVP	3500	3637	+137
4F'	B3LYP-D3(BJ)	def2-TZVP	3502	3552	+50
4F'	B3LYP-D3(BJ)	ma-def2-TZVP	3500	3555	+55
0F	B3LYP-D3(BJ)	def2-TZVP	3491	3547	+55
0F	B3LYP-D3(BJ)	ma-def2-TZVP	3491	3547	+56

Table S6: Calculated infrared band strength σ in $\mathrm{kJ\,mol^{-1}}$ for each docking side and corresponding infrared band strength ratios $\frac{\sigma_{\mathrm{Ph}}}{\sigma_{\mathrm{Me}}}$. Aromatic stacking correlates with a low visibility of the phenyl side docking isomer due to a weakening of the hydrogen bond interaction.

Acceptor	Method	Basis set	σ_{Ph}	σ_{Me}	$\frac{\sigma_{\mathrm{Ph}}}{\sigma_{\mathrm{Me}}}$
2Br	B3LYP-D3(BJ)	def2-TZVP	518	1039	0.499
2Br	B3LYP-D3(BJ)	ma-def2-TZVP	525	1025	0.512
2Cl	B3LYP-D3(BJ)	def2-TZVP	519	1081	0.480
2Cl	B3LYP-D3(BJ)	ma-def2-TZVP	530	1083	0.490
2F	B3LYP-D3(BJ)	def2-TZVP	277	1225	0.226
2F	B3LYP-D3(BJ)	ma-def2-TZVP	280	1245	0.225
2F’	B3LYP-D3(BJ)	def2-TZVP	570	1225	0.465
2F’	B3LYP-D3(BJ)	ma-def2-TZVP	575	1245	0.462
4Br	B3LYP-D3(BJ)	def2-TZVP	178	1272	0.140
4Br	B3LYP-D3(BJ)	ma-def2-TZVP	182	1311	0.139
4Cl	B3LYP-D3(BJ)	def2-TZVP	184	1268	0.145
4Cl	B3LYP-D3(BJ)	ma-def2-TZVP	183	1295	0.141
4F	B3LYP-D3(BJ)	def2-TZVP	206	1208	0.171
4F	B3LYP-D3(BJ)	ma-def2-TZVP	207	1235	0.168
4F’	B3LYP-D3(BJ)	def2-TZVP	770	1208	0.637
4F’	B3LYP-D3(BJ)	ma-def2-TZVP	752	1235	0.609
0F	B3LYP-D3(BJ)	def2-TZVP	813	1272	0.639
0F	B3LYP-D3(BJ)	ma-def2-TZVP	804	1281	0.628

Experimental Results

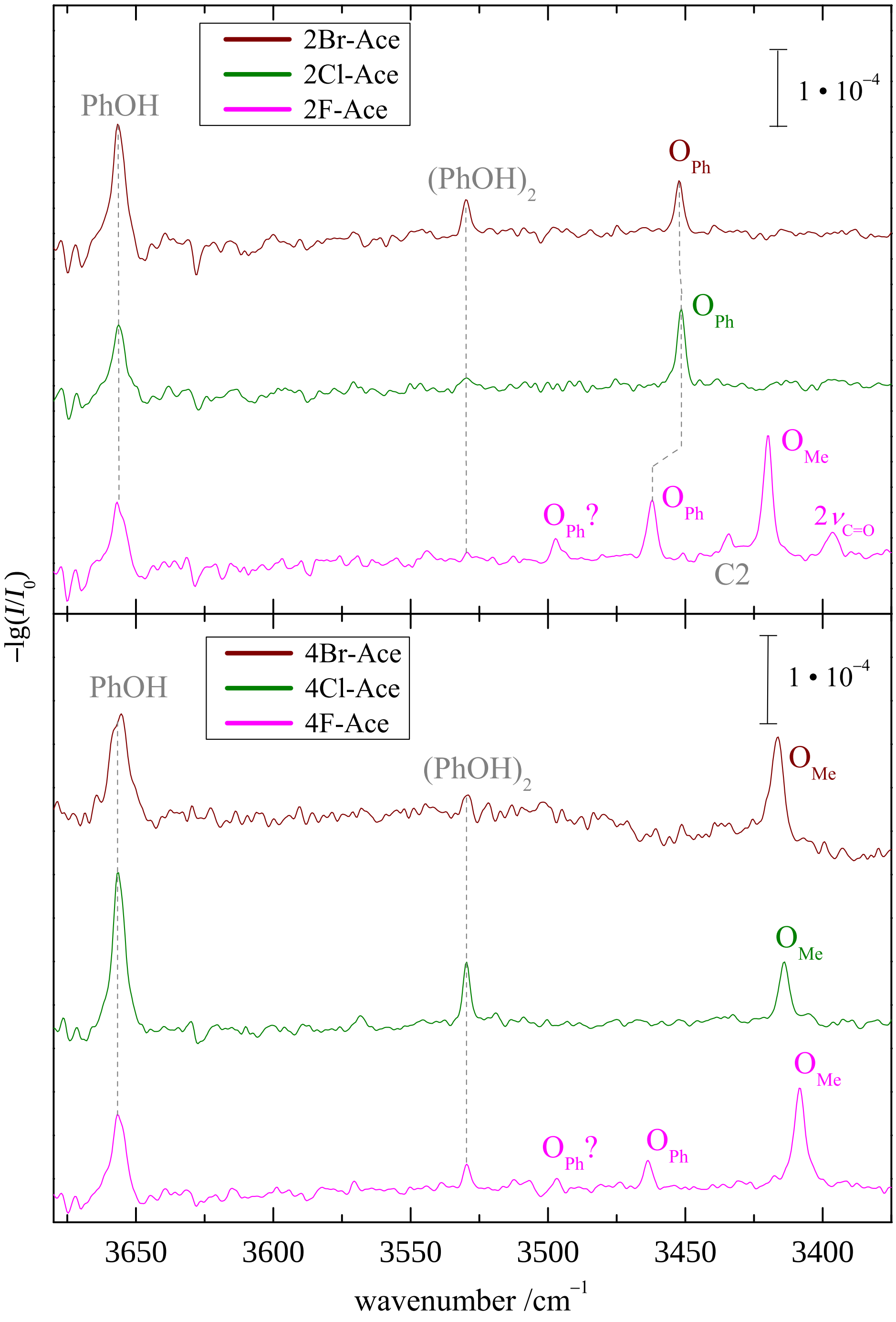

Fig. S3: FTIR jet OH stretching spectra of phenol with the corresponding acetophenone derivative. The 1:1 complexes are marked with O, indexed by the assigned docking preference (Ph for phenyl docking and Me for methyl docking). Upper panel: Spectra for phenol with acetophenone derivatives substituted at the second position in the acetophenone ring. C1 and C2 mark higher clusters and $2\nu_{C=O}$ marks the overtone of the C=O stretching frequency. Lower panel: Spectra for phenol with acetophenone derivatives substituted at the fourth position in the acetophenone ring.

Table S7: Experimental OH stretching wavenumbers $\tilde{\nu}$, resulting wavenumber shifts $\Delta\tilde{\nu}_{\mathrm{h-l}}$ for high (h) and low (l) lying as well as uncertain (O?) spectral signals for phenol-acetophenone derivative 1:1 complexes and $2\tilde{\nu}_{\mathrm{C=O}}$ C=O stretch overtone of the corresponding monomeric ketone in cm^{-1}. The accidental overlap of the monomeric C=O stretch overtone with the OH stretch fundamental of the methyl side docking isomer of the complex for 0F and 4F requires the assessment of potential intensity distortions before analyzing integrated intensity ratios.

Acceptor	$\tilde{\nu}_{\mathrm{O?}}$	$\tilde{\nu}_{\mathrm{high}}$	$\tilde{\nu}_{\mathrm{low}}$	$\Delta\tilde{\nu}_{\mathrm{O?-low}}$	$\Delta\tilde{\nu}_{\mathrm{high-low}}$	$2\tilde{\nu}_{\mathrm{C=O}}$
2Br	-	3452	-	-	-	-
2Cl	-	3451	-	-	-	-
2F	3497	3462	3420	77	43	3397
4Br	3502	-	3417	85	-	-
4Cl	-	-	3414	-	-	-
4F	3497	3464	3409	89	55	3404
0F	-	3458	3400	-	58	3402

Table S8: Experimental ranges for the integrated intensity ratios $I_{\mathrm{Me}}/I_{\mathrm{Ph}}$. Together with absorption cross-sections from theory (from two different basis sets), docking ratios $c_{\mathrm{Me}}/c_{\mathrm{Ph}}$ and resulting experimental fractions x_{Ph} for Ph docking are derived. Ranges for $I_{\mathrm{Me}}/I_{\mathrm{Ph}}$ are obtained using an automated statistical evaluation[S4] and assuming exact theoretical cross section ratios for two observed bands. Bounds for $I_{\mathrm{Me}}/I_{\mathrm{Ph}}$ are obtained from single bands and from the spectral noise hiding a hypothetical second band, using a quantile difference $q_{97.5} - q_{5.0}$ of the obtained distribution.[S5] Where both estimates are available because a band is questionable (2F, 4F), the more generous bound (in bold face) is chosen in the main text.

Basis set	Acceptor	Consid. bands	$\frac{I_{\mathrm{Me}}}{I_{\mathrm{Ph}}}$	$\frac{c_{\mathrm{Me}}}{c_{\mathrm{Ph}}}$	x_{Ph}
def2-TZVP	2Br	O_{Ph}	< 0.27	< 0.14	> 0.88
	2Cl	O_{Ph}	< 0.25	< 0.12	> 0.89
	2F'	O_{Me},O_{Ph}	1.47-2.33	0.68-1.08	0.48-0.59
	2F	O_{Me},O_{Ph}?	**4.39**-10.84	**0.99**-2.45	0.29-**0.50**
		or only O_{Me}	> 4.65	> 1.05	< 0.49
	4Br	O_{Me}	> 2.73	> 0.38	< 0.72
	4Cl	O_{Me}	> 4.23	> 0.61	< 0.62
	4F'	O_{Me},O_{Ph}	3.67-6.83	2.34-4.35	0.19-0.30
	4F	O_{Me},O_{Ph}?	8.40-13.60	1.43-2.32	0.30-0.41
		or only O_{Me}	**> 5.42**	**> 0.92**	**< 0.52**
	0F	O_{Me},O_{Ph}	**6.58**-12.80	**4.20**-8.18	0.11-**0.19**
		or only O_{Me}	> 8.48	> 5.42	< 0.16
ma-def2TZVP	2Br	O_{Ph}	< 0.27	< 0.14	> 0.88
	2Cl	O_{Ph}	< 0.25	< 0.12	> 0.89
	2F'	O_{Me},O_{Ph}	1.47-2.33	0.68-1.08	0.48-0.60
	2F	O_{Me},O_{Ph}?	**4.39**-10.84	**0.99**-2.44	0.29-**0.50**
		or only O_{Me}	> 4.65	> 1.05	< 0.49
	4Br	O_{Me}	> 2.73	> 0.38	< 0.73
	4Cl	O_{Me}	> 4.23	> 0.60	< 0.63
	4F'	O_{Me},O_{Ph}	3.67-6.83	2.24-4.16	0.19-0.31
	4F	O_{Me},O_{Ph}?	8.40-13.60	1.41-2.28	0.30-0.41
		or only O_{Me}	**> 5.42**	**> 0.91**	**< 0.52**
	0F	O_{Me},O_{Ph}	**6.58**-12.80	**4.13**-8.04	0.11-**0.19**
		or only O_{Me}	> 8.48	> 5.33	< 0.16

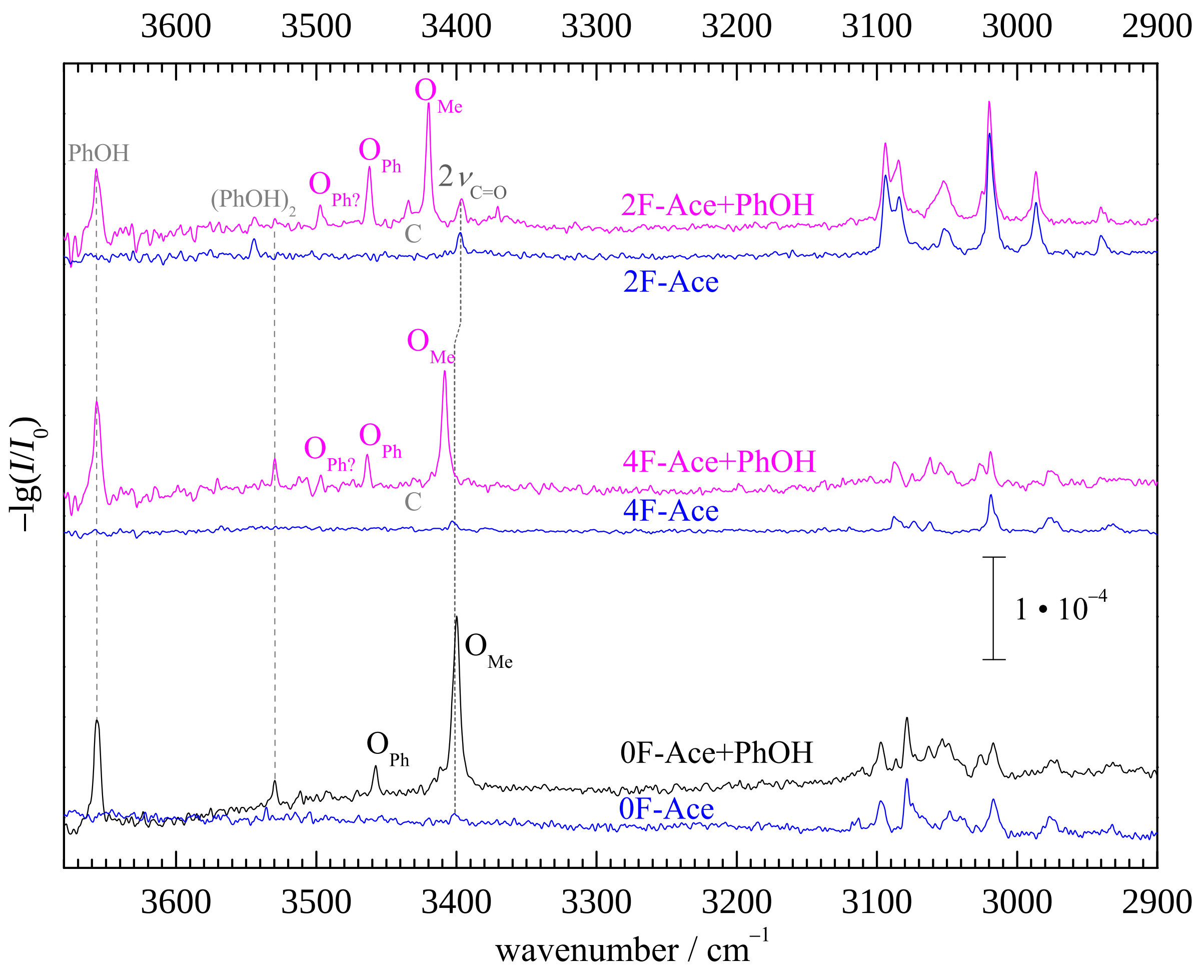

Fig. S4: FTIR jet OH stretching spectra of 2F-, 4F- and 0F-acetophenone with phenol compared to corresponding (monomer-scaled) pure ketone spectra (blue). $2\nu_{\mathrm{C=O}}$ marks the overtone of the C=O stretching frequency around $3400\,\mathrm{cm}^{-1}$ and dashed lines connect its substitution-dependent positions as well as the phenol monomer and dimer[S6] positions. For 2F, $2\nu_{\mathrm{C=O}}$ does not overlap with the mixed dimer band $\mathrm{O_{Me}}$, in contrast to 4F and 0F. There, the contributions are comparable to the noise in the mixed spectra and can therefore be neglected.

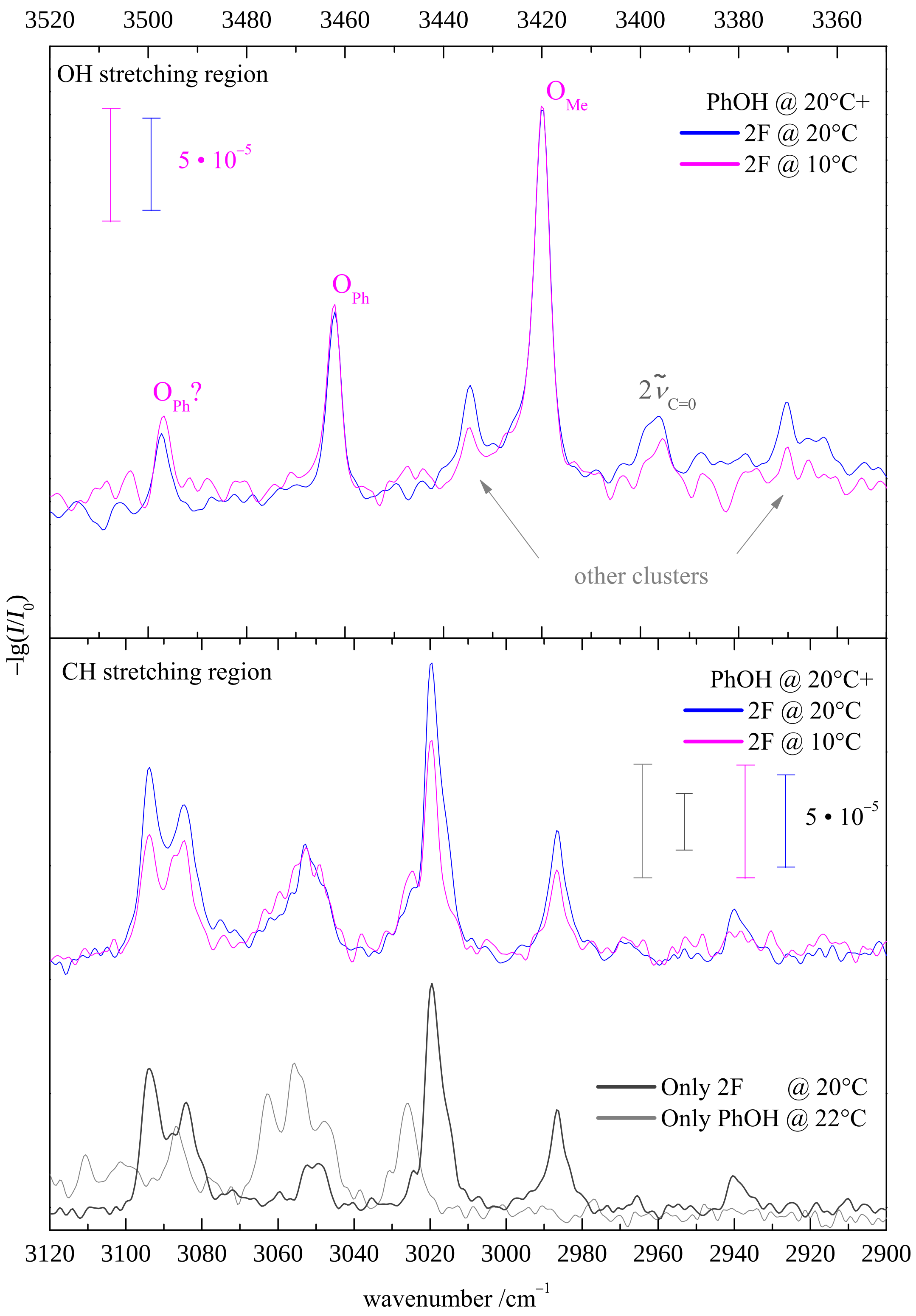

Fig. S5: FTIR jet OH stretching spectra of phenol (upper panel) in the presence of different 2F concentrations, scaled to match the O_{Me} intensity of the 1:1 complex. This hints at other cluster compositions (peaks without such a match, see arrows), whereas O_{Ph} and less certain O_{Ph}? are suggested to be due to 1:1 complexes. For the confirmation of additional cluster contributions, the CH stretching region (lower panel) of the two scaled spectra and of two mono-component spectra (2F and PhOH only) is shown.

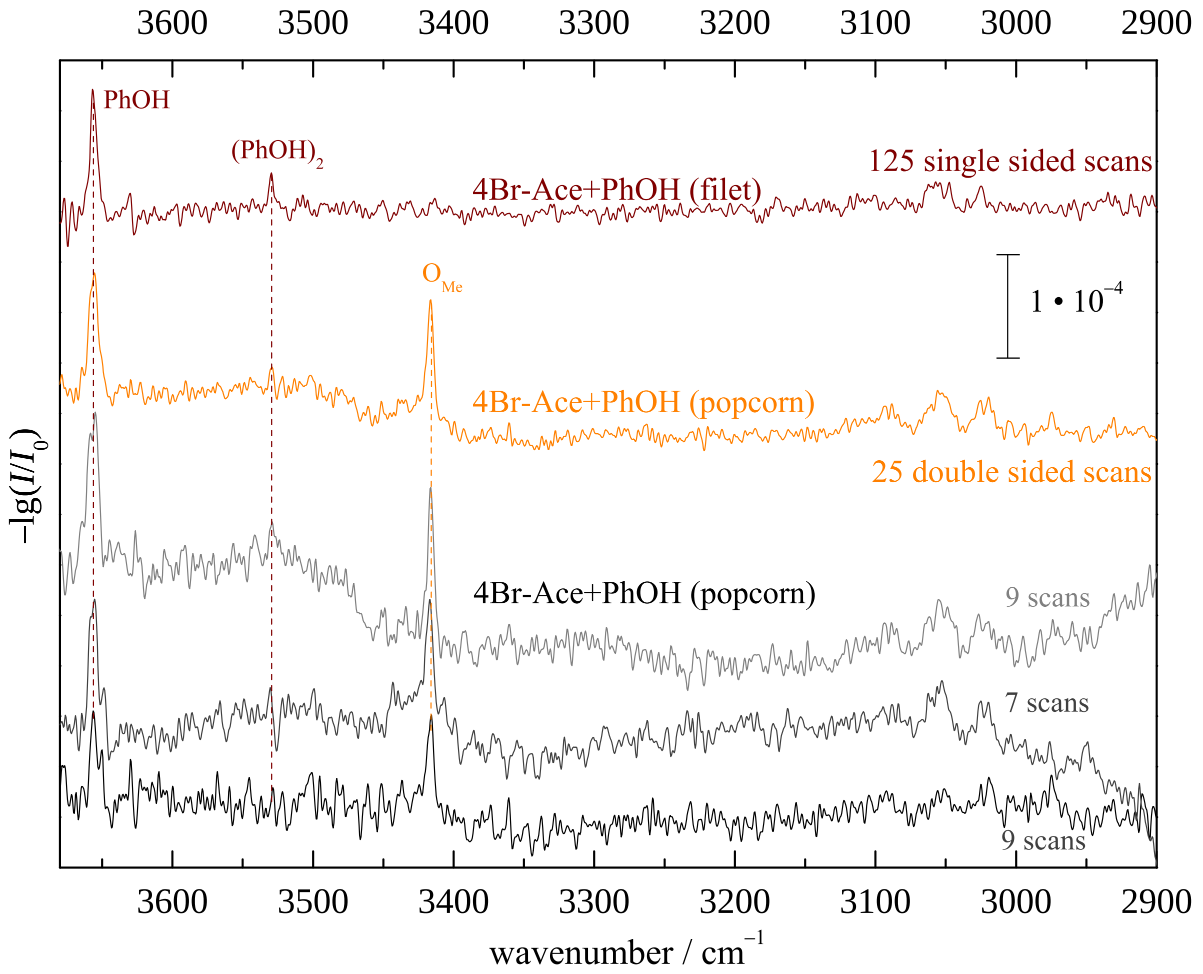

Fig. S6: The heatable nozzle spectrometer[S7,S8] was modified to allow for two compounds of low (and different) volatility, in this case phenol and 4Br-acetophenone. The carrier gas pulse (200 ms) is led through two (instead of one) heatable sample chambers in sequence, enclosed by poppet valves P, leading to the flow sequence (P1=1 psi | phenol | P2=1 psi | 4Br-acetophenone | P3=5 psi | nozzle). All zones can be heated individually in a range from 310 K to 470 K but influence each other for large temperature differences, leading to concentration drifts over time. Therefore, only 25 pulses with appropriate temperature settings and similar concentrations from three different measurements were obtained (orange spectrum). The temperature sequences of these three measurements were as follows: (P1=313 K | phenol=335 K | P2=373 K | 4Br-acetophenone=363 K | P3=383 K | nozzle=383 K) (light grey and black spectrum with 9 scans each) and (P1=333 K | phenol=318 K | P2=353 K | 4Br-acetophenone=393 K | P3=413 K | nozzle=413 K) (dark grey spectrum with 7 scans) respectively. The enhanced vapor pressure compensates in part for the low number of pulses, as the comparison of the spectra obtained with the 600 mm room temperature nozzle ("filet-jet") and the 60 mm heatable nozzle ("popcorn-jet") shows. However, contributions from larger clusters cannot be ruled out as safely as for the room temperature expansion.

References

(S1) Neese, F. Software update: the ORCA program system, version 4.0. *WIREs Comput. Mol. Sci.* **2018**, *8*, e1327.

(S2) TURBOMOLE V7.3 2018, a development of University of Karlsruhe and Forschungszentrum Karlsruhe GmbH, 1989-2007, TURBOMOLE GmbH, since 2007; available from `http://www.turbomole.com`.

(S3) Furche, F.; Ahlrichs, R.; Hättig, C.; Klopper, W.; Sierka, M.; Weigend, F. Turbomole. *WIREs Comput. Mol. Sci.* **2014**, *4*, 91–100.

(S4) Karir, G.; Lüttschwager, N. O. B.; Suhm, M. A. Phenylacetylene as a gas phase sliding balance for solvating alcohols. *Phys. Chem. Chem. Phys.* **2019**, *21*, 7831–7840.

(S5) Lüttschwager, N. O. B. NoisySignalIntegration.jl: A Julia package to determine uncertainty in numeric integrals of noisy x-y data. Available from GitHub.com: `https://github.com/nluetts/NoisySignalIntegration.jl`.

(S6) Ebata, T.; Watanabe, T.; Mikami, N. Evidence for the Cyclic Form of Phenol Trimer: Vibrational Spectroscopy of the OH Stretching Vibrations of Jet-Cooled Phenol Dimer and Trimer. *The Journal of Physical Chemistry* **1995**, *99*, 5761–5764.

(S7) Altnöder, J.; Lee, J. J.; Otto, K. E.; Suhm, M. A. Molecular Recognition in Glycolaldehyde, the Simplest Sugar: Two Isolated Hydrogen Bonds Win Over One Cooperative Pair. *ChemistryOpen* **2012**, *1*, 269–275.

(S8) Hartwig, B.; Lange, M.; Poblotzki, A.; Medel, R.; Zehnacker, A.; Suhm, M. A. The reduced cohesion of homoconfigurational 1,2-diols. *Phys. Chem. Chem. Phys.* **2020**, *22*, 1122–1136.
